# Pretreatment-dependent surface chemistry of wood nanocellulose for pH-sensitive hydrogels

**DOI:** 10.1177/0885328214531511

**Published:** 2014-09

**Authors:** Gary Chinga-Carrasco, Kristin Syverud

**Affiliations:** Paper and Fibre Research Institute (PFI) – Høgskoleringen 6b, Trondheim, Norway

**Keywords:** Nanocellulose, nanofibrillated cellulose, hydrogels, cross-linking, pH responsive, characterisation, wound healing

## Abstract

Nanocellulose from wood is a promising material with potential in various technological areas. Within biomedical applications, nanocellulose has been proposed as a suitable nano-material for wound dressings. This is based on the capability of the material to self-assemble into 3D micro-porous structures, which among others have an excellent capacity of maintaining a moist environment. In addition, the surface chemistry of nanocellulose is suitable for various applications. First, OH-groups are abundant in nanocellulose materials, making the material strongly hydrophilic. Second, the surface chemistry can be modified, introducing aldehyde and carboxyl groups, which have major potential for surface functionalization. In this study, we demonstrate the production of nanocellulose with tailor-made surface chemistry, by pre-treating the raw cellulose fibres with carboxymethylation and periodate oxidation. The pre-treatments yielded a highly nanofibrillated material, with significant amounts of aldehyde and carboxyl groups. Importantly, the poly-anionic surface of the oxidized nanocellulose opens up for novel applications, i.e. micro-porous materials with pH-responsive characteristics. This is due to the swelling capacity of the 3D micro-porous structures, which have ionisable functional groups. In this study, we demonstrated that nanocellulose gels have a significantly higher swelling degree in neutral and alkaline conditions, compared to an acid environment (pH 3). Such a capability can potentially be applied in chronic wounds for controlled and intelligent release of antibacterial components into biofilms.

## Introduction

Nanocellulose is a novel material that can be produced from wood, non-woody plants and agricultural residues.^1–5^ The morphology and type of wood nanocellulose may vary significantly, including cellulose nanocrystals, micro- and nanofibrillated celluloses. Nanofibrillated cellulose has commonly been produced with homogenizers, fluidizers and grinders.^4,6–8^ Nanofibrillated cellulose materials contain a major fraction of nanofibrils with typical diameters less than 20 nm and lengths in the micrometre scale.^9^

Pre-treatments, which are applied to facilitate the fibrillation of cellulose fibres, involve mechanical, enzymatic and chemical pre-treatments.^1,2,10^ Mechanical and enzymatic pre-treatments preserve the surface chemistry of cellulose fibres, i.e. the surface of cellulose nanofibrils is strongly hydrophilic and is covered by hydroxyl groups. Chemical pre-treatments yield a homogeneous nanofibril morphology, with typical widths less than 20 nm.^1,2,11^ TEMPO-mediated oxidation leads to a regiospecific introduction of carboxyl groups in the C6 position and small amounts of aldehyde groups.^1^ Carboxymethylation modifies the surface chemistry of the nanofibrils, introducing carboxymethyl groups.^2^ In addition to the mentioned chemical pre-treatments,^1,2^ sodium metaperiodate, followed by an oxidation with sodium chlorite, has been applied to enhance the nanofibrillation of cellulose fibres^12^ and reduce energy consumption during production of nanocellulose.^13^ Sodium metaperiodate oxidizes cellulose by selective cleavage between the C2 and C3 groups and yields 2,3-dialdehyde units along the polymer chain.^14^

In addition to controlling the nanofibril morphology, the design of the corresponding surface chemistry is most interesting from various points of view. First, the aldehyde groups can react with amines and be used for polymer–protein conjugation by covalent binding. Second, the carboxyl groups can be used for reaction with e.g. divalent cations or surfactants by ionic interactions. Hence, careful design of the pre-treatment can yield a tailor-made surface chemistry, which can react with cationic polymers, nanoparticles and relevant biomolecules. Such surface modification can be the basis for adding functionality to the material. As a concrete example, oxidized cellulose nanofibrils can form hydrogels,^15^ which can have a range of applications within the biomedical and pharmaceutical fields. This is motivated by the capability of hydrogels to respond to physiological stimuli,^16^ by e.g. swelling. The swelling capacity of a polymer network depends on e.g. polymer solvent compatibility, the degree of cross-linking and the ionic interaction.^17^ A controlled swelling of nanocellulose 3D networks offers a unique opportunity to apply this novel material in wound management.

Various studies have confirmed that nanocellulose materials from wood are not cytotoxic. The tests have been performed using a series of different cell lines and different nanocelluloses, including cellulose nanocrystals, micro- and nanofibrillated celluloses.^18–20^ Hence, nanocellulose from wood pulp fibres is a promising alternative to bacterial cellulose as a biomaterial and specifically for the manufacturing of wound dressings. Importantly, compared to the production of bacterial cellulose, which can be difficult to scale-up,^21^ nanocellulose from wood can be produced in large scale and with tailor-made morphology and surface chemistry.

The purpose of this study was to produce nanocellulose from wood with controlled surface chemistry and morphology, which could potentially be exploited to add functionality to the material. Based on neat and cross-linked nanofibrillated structures, the swelling and pH-responsive behaviour of the oxidized nanocellulose hydrogels were assessed.

## Materials and methods

### Nanocellulose production

Never-dried bleached *Pinus radiata* market pulp fibres were used in this study. The pulp fibres were pre-treated before homogenization. The pre-treatment comprised three different procedures: (a) carboxymethylation, (b) carboxymethylation and periodate and (c) periodate oxidation. It was expected that the pre-treatments applied in this study, which involved carboxymethylation, would produce highly nanofibrillated qualities, which we will refer to as nanocellulose in the following.

#### Carboxymethylation (sample CMNC)

The pulp was pre-treated using a method for low degree of substitution carboxymethyl cellulose, suggested by Walecka.^22^ The specific conditions are described in Wågberg et al.^2^ About 110 g of the pulp was disintegrated according to ISO standard 5263-1.^23^ This was followed by exchange of solvent from water to ethanol. The fibres were impregnated in a solution of 2% monochloroacetic acid in 500 mL isopropanol for 30 min. This was transferred to a 5-L reaction vessel equipped with reflux and containing a heated solution of 16.2 g NaOH in a mixture of 500 mL methanol and 2 L isopropanol. The carboxymethylation reaction was allowed to continue for 1 h. The carboxymethylated pulp was washed with 20-L deionized water and 2-L 0.1 M acetic acid followed by additional 10-L deionized water. The carboxyl groups were converted to sodium form by soaking the pulp in a 4% NaHCO_3_ solution for 60 min. The pulp was finally filtered and washed with deionized water until the conductivity was <5 µS/cm.

#### Periodate oxidation (sample PNC)

The reaction was performed in a 4% suspension of cellulose pulp. About 0.82 g NaIO_4_ was added per gram cellulose. The NaIO_4_ was dissolved before the cellulose pulp was added. The NaIO_4_ solution was protected against light by covering the reaction vessel with an aluminium foil. The reaction was allowed to continue for 2 h at 55℃. The pulp was filtered and washed with deionized water until the conductivity was <5 µS/cm. For details, see Shet^24^ and Sirvio et al.^25^

#### Carboxymethylation and periodate treatment in combination (sample CMPNC)

The pulp fibres were both carboxymethylated and oxidized using NaIO_4_ according to the methods described above. Carboxymethylation was performed prior to NaIO_4_ oxidation.

The pre-treated pulp fibres were then homogenized with a Rannie 15 type 12.56 × homogenizer. The pulp consistency during the homogenization was 0.5%. The carboxymethylated, carboxymethylated and periodate, and periodate pre-treated nanocelluloses will be referred to as sample CMNC, CMPNC and PNC, respectively.

### Degree of polymerization and surface chemistry

The degree of polymerization (DP) was calculated from intrinsic viscosity values determined according to the standard ISO 5351:2010.^26^ The DP was assessed before the homogenization of the pre-treated pulp fibres. In order to prevent cleavage of the cellulose polymer caused by the presence of aldehyde groups, these groups were further oxidized to carboxyl groups before the dissolution in the copper ethylenediamine solution, as described by Shinoda et al.^27^ This was done by the addition of 1.81 g NaClO_2_ to 2-g pre-treated pulp in a 2% suspension of 1 M CH_3_COOH. The oxidation was carried out by stirring the mixture at room temperature for 48 h, as described by Saito and Isogai.^28^ The DP was calculated using the following equation: DP^0.905 ^= 0.75 [η].^29^

The content of carboxyl and aldehyde groups was determined by conductometric titration, as described by Saito and Isogai.^28^ About 55 mL water and 5 mL 0.01 M NaCl were added to 0.3 g dry sample. The pH was adjusted to approximately 2.5 by the addition of 0.1 M HCl. Titration was performed with 0.04 M NaOH solution added at a rate of 0.1 mL/min up to pH of approximately 11 using an automatic titrator (Metrohm 902 Titrando). The conductivity of the sample was automatically measured at increments of 0.01 mL using a Metrohm 856 Conductivity Module, and the data was recorded by Tiamo® Titration Software. The carboxylate content was calculated from the titration curve (Gran plot). This analysis was also done after oxidation of aldehyde groups to carboxyl groups with NaClO_2_ (procedure described above). The difference in carboxylate content before and after the NaClO_2_ oxidation yields the aldehyde content.

### Structural and optical assessment of films

Ten films were made by dispersion casting on petri dishes, from the three nanocellulose samples, i.e. CMNC, CMPNC and PNC. The grammage of each film was 20 g/m^2^. The films were allowed to dry in room temperature. After drying, it was not possible to remove the sample PNC from the petri dish surface. Hence, films of sample PNC were additionally made on plastic films in order to perform the corresponding structural and optical analyses.

Ten laser profilometry (LP) topography images were acquired from the top sides of each film sample. The lateral and z-resolution of the LP system was 1 µm and 10 nm, respectively. The size of the local areas was 1 mm × 1 mm. The surfaces were horizontally levelled. The surface images were bandpass filtered for quantifying the surface topography (Sq) at wavelengths below 160 µm.

AFM imaging was performed with a Multimode AFM (with Nanoscope V controller), Digital Instruments. Four images from each sample were recorded in ScanAsyst mode at room temperature in air. The AFM tips of spring constant value ∼0.4 N/m were purchased from Bruker AFM probes. The size of the assessed areas was 2 µm × 2 µm. The image size was 1024 × 1024 pixels. For details on the structural micro- and nano-surface analyses, see Chinga-Carrasco et al.^30^ In addition, the nanofibril width distribution was calculated based on automatic image analysis of the AFM images.^11^

UV–vis transmittance was quantified with a UV–visible spectrophotometer (Cary 300 Conc, Varian). The wavelengths between 200 and 800 nm were included for analysis. Three replicates were undertaken from each sample.

### Hydrogel characteristics

The gels (CMNC, CMPNC, PNC) were cast in metal moulds (height = 18 mm, diameter = 16 mm). The samples were freeze-dried by freezing at −18℃ and dried for 2 days in a Heto, LyoPro 6000 equipment. In addition, the CMPNC sample was cross-linked with hexamethylenediamine (HMDA) at a concentration of 1200 µmol/g. After mixing and casting the CMPNC sample with HMDA, the moulds were put in an oven at 80℃ overnight before freeze-drying.

In addition, in order to confirm the reaction between carboxyl groups and divalent cations, the CMNC and CMPNC gels were mixed with a CaCl_2_ solution (5 mmol/g) to form a Ca^2+^-nanocellulose composite gel. The suspensions were freeze-dried, as described above.

The moisture absorption was measured as a function of time at intervals 15, 30, 60, 120, 180 and 240 min. After each interval, the hydrogels were removed from the aqueous medium, slightly dried with a filter paper to remove surplus water and weighed. Additionally, the swelling of the neat CMNC and cross-linked CMPNC hydrogels were assessed after 120 min in water at pH 3, 7 and 9. The pH was adjusted with HCl and NaOH.

The swelling capacity of the hydrogels was estimated using equation (1).

(1) $$\mathrm{swelling}(\%)=\frac{\left(\mathrm{Ws}-\mathrm{Wd}\right)}{\mathrm{Wd}}\times 100$$

where *Ws* and *Wd* correspond to the weight of the wetted and dry sample, respectively.

The freeze-dried micro-porous structures were analyzed with field-emission scanning electron microscopy. The samples were coated with a layer of gold. The applied microscope was a Zeiss Ultra field-emission SEM (Zeiss Ultra field-emission SEM, Carl Zeiss AG, Oberkochen, Germany). Images were acquired in secondary electron imaging mode. The acceleration voltage and working distance were 5 kV and 13 mm, respectively.

## Results and discussion

### Quantification of nanocellulose structural characteristics

**Figure 1.**
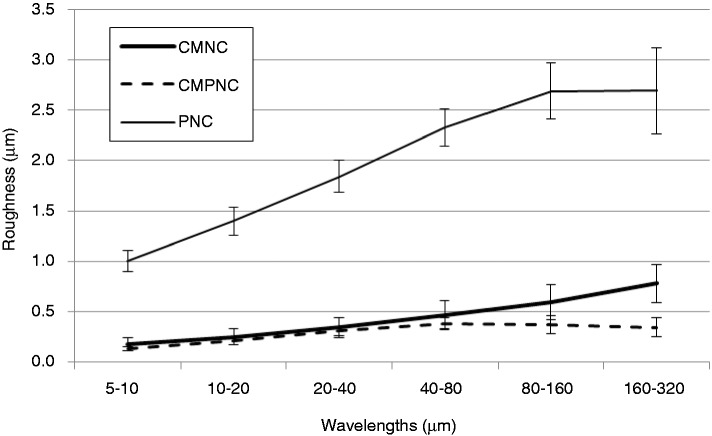
LP roughness of nanocellulose films, assessed at several wavelengths. The carboxymethylated (CMNC), carboxymethylated and periodate (CMPNC) and periodate oxidated (PNC) samples are included. The results are given with ±1 STD.

The surface micro- and nano-structural characteristics of the films were assessed by LP and AFM, respectively. The surfaces of the films from samples CMNC and CMPNC were smooth, compared to a rough structure of the sample pre-treated with periodate only (sample PNC). The differences are clearly confirmed by a multi-scale roughness quantification, which indicates that the sample CMPNC is relatively smooth at all the assessed wavelengths (Figure 1), thus indicating a highly nanofibrillated material.^30^

**Figure 2.**
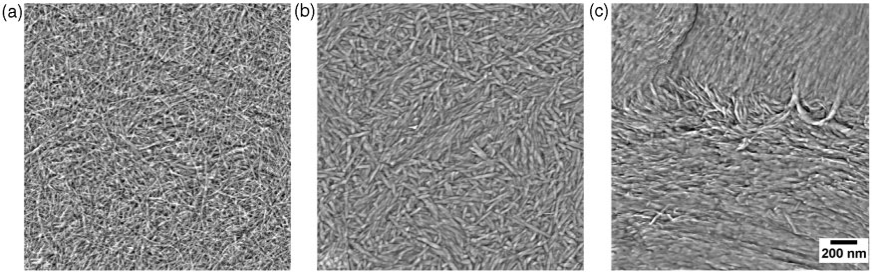
AFM characterisation. (a) Carboxymethylated sample (CMNC); (b) carboxymethylated and periodate pre-treated sample (CMPNC); (c) periodate pre-treated sample (PNC). The images have been enhanced to improve the visualization of the nanofibrils.

**Figure 3.**
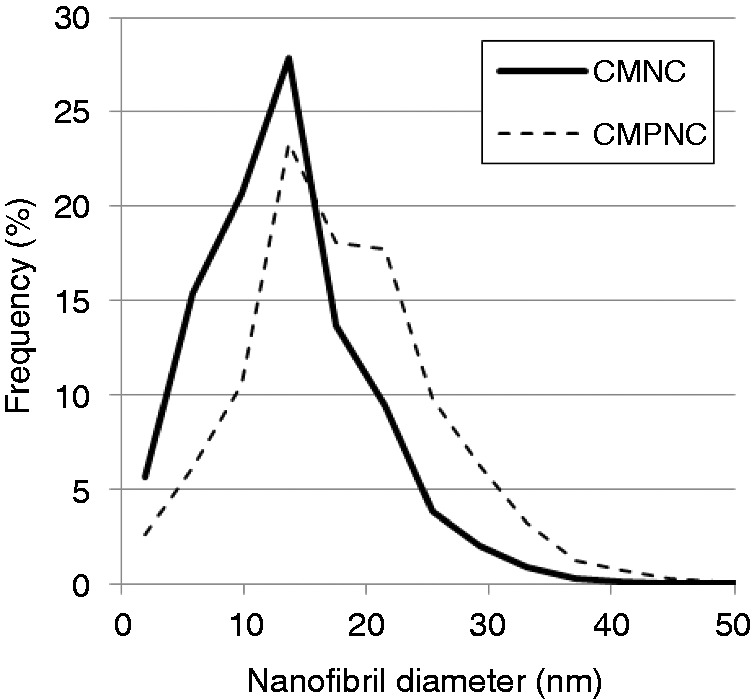
Nanofibril diameter distribution based on AFM images and automatic image analysis.

Assessment of the surface microstructure was complemented with a detailed assessment of the surface nano-structure with AFM (Figure 2). Note that the CMNC-sample has a major fraction of cellulose nanofibrils with average width 15 nm (±0.8 nm). Interestingly, the CMPNC-sample seems to be composed of relatively thicker and apparently shorter nanofibrils (Figure 2(b)). The average width of the CMPNC sample is 20 nm (±0.8 nm) as quantified automatically by image analysis of the AFM images. The nanofibril width distribution of the CMNC and CMPNC samples is given in Figure 3. Care must be taken when quantifying the width distribution of nanofibrils with AFM as the lateral dimensions may be overestimated depending on the used AFM tip. In this study, the thicker morphology of the nanofibrils is in accordance with the quantification performed by Liimatainen et al.,^12^ who reported nanofibril widths of approximately 25 nm (±6 nm). Note that individualized nanofibrils yielded by e.g. TEMPO-mediated oxidation or carboxymethylation have been reported to have widths of less than 20 nm.^2,11,31–33^

The PNC-sample had a relatively rougher morphology, compared to the CMNC and CMPNC samples. The AFM roughness values (areas 2 × 2 µm) of the samples CMNC, CMPNC and PNC were 11.3 (±7.2), 4.0 (±0.36) and 68.3 (±44.4) nm, respectively. The LP and AFM analyses confirm the smooth surface characteristics of the CMPNC sample and thus the high degree of fibrillation of the material.

**Figure 4.**
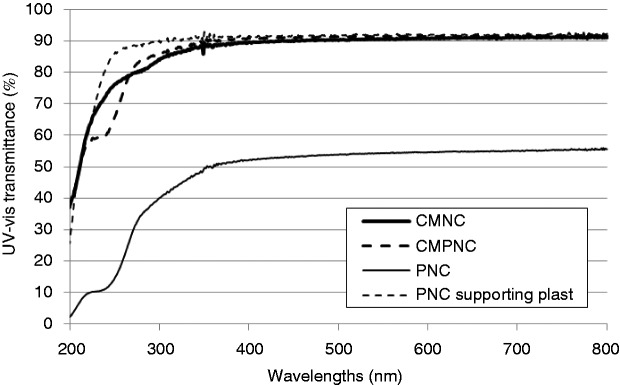
UV–vis transmittance of samples CMNC, CMPNC and PNC. The transmittance curve of the plastic film used for supporting the PNC sample is included as a reference.

The UV light transmittance of the assessed films confirms the fibrillation degree of the produced nanocelluloses (Figure 4). The light transmittance at a wavelength of 600 nm was 91% for samples CMNC and CMPNC, confirming the translucency of the films due to a high-fibrillation degree of the nanocellulose materials.^34–36^ The transmittance of sample PNC is 55% (at 600 nm wavelength) due to the coarse structure of the fibrous material. Note the “shoulder” at bands between 200 and 300 nm of samples CMPNC and PNC, which are indications of the occurrence of aldehyde groups.^35^ Such shoulders are not detected in the nanocellulose sample produced with carboxymethylation as pre-treatment (sample CMNC), which confirms this observation.

### Surface chemistry and DP

Table 1 shows the DP and amounts of aldehyde and carboxyl groups. Note the low DP of the periodate pre-treated samples (CMPNC and PNC, Table 1). Keep in mind that the periodate pre-treatment introduces a cleavage between the C2 and C3 groups,^14^ which may weaken and shorten the cellulose polymers. The low DP seems to agree with the apparently shorter nanofibrils observed in the AFM analysis (Figure 2). Fukuzumi et al.^37^ also reported a correlation between the DP and the length of TEMPO-mediated oxidized nanofibrils, which became shorter in association with depolymerisation.

**Table 1.** DP, aldehyde and carboxyl content of samples CMNC and CMPNC.

	DP	Aldehyde content (µmol/g)	Carboxyl content (µmol/g)
CMNC	950	12	440
CMPNC	<80	1202	393
PNC	<70	1410	25

As expected, the CMNC and the PNC pre-treated samples contain a significant amount of carboxyl and aldehyde groups, respectively. Additionally, the combination of carboxymethylation and periodate oxidation yields nanofibrils with significant amount of both carboxyl and aldehyde groups, which confirms a successful pre-treatment. Periodate oxidation yields 2,3-dialdehydes along the cellulose polymer.^14^ Note the aldehyde content of samples CMPNC and PNC, achieving levels of >1200 µmol/g. This is a significant increment compared to e.g. TEMPO-mediated oxidation, where levels of <200 µmol/g have been reported.^38,39^ Liimatainen et al.^12^ used periodate and chlorite oxidation for facilitating the fibrillation of cellulose pulp fibres. As exemplified in Figures 1, 2 and 4, periodate alone does not seem to facilitate the production of a nanofibrillated structure. The charge repulsion introduced by carboxyl groups is necessary to promote fibrillation,^12^ which is confirmed in this study. Important to mention, Liimatainen et al.^12^ did not attempt to control the surface chemistry of nanocellulose, as the formed aldehyde groups were totally oxidized into carboxyl groups after chlorite oxidation.

It is important to note that the aldehyde groups can react with amines^15^ and be used for polymer–protein conjugation by covalent binding. Such a capability can be exploited for adding functionality to nanocellulose structures. In addition, carboxyl groups, which can function as ionisable functional groups, can be utilized for making pH-responsive hydrogels.^40^ This will be explored in the following section.

### Hydrogels characteristics

**Figure 5.**
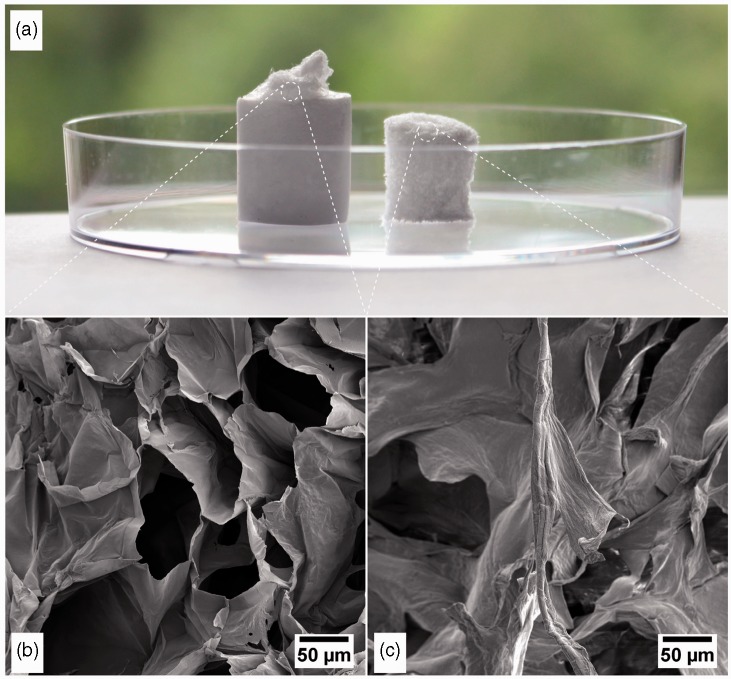
Freeze-dried structures of carboxymethylated pre-treated samples. (a) The freeze-dried structures without (left) and with Ca^2+^ (right) are exemplified. (b) FESEM of the micro-porous structure of the carboxymethylated nanocellulose sample. (c) FESEM of the micro-porous structure of the carboxymethylated nanocellulose, which is ionic-bonded with Ca^2+^. FESEM: field-emission scanning electron microscopy.

According to Czaja et al.,^21^ the creation of a moist environment, the ability to control fluid loss and the absorption of exudates are important characteristics of a modern wound dressing. Such characteristics can be related to the porous structure and surface chemistry of the material. The porous structure of the CMNC sample is exemplified in Figure 5. Note the structure composed of pores in the micrometer scale (Figure 5(b) and (c)). The pores are defined by walls, which are composed of self-assembled cellulose nanofibrils.^15^ The CMNC sample, which was made in the presence of Ca^2+^, was smaller and apparently denser than the neat CMNC sample (Figure 5(a)). This is most probably due to the ionic links between the COO^−^ groups and the Ca^2+^, which increased the cross-linking density and reduced the swelling capacity of the gel.

**Figure 6.**
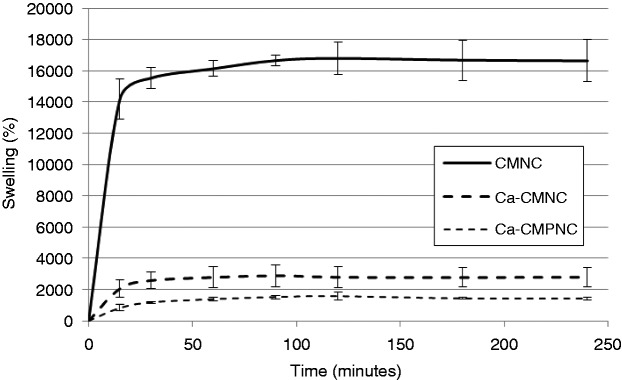
Swelling of nanocellulose hydrogels. The Ca^2+^-CMNC and Ca^2+^-CMPNC samples are included for comparison. The results are given with ±1 STD.

Quantification of the swelling (equation (1)) reveals a great capacity of the nanocellulose material to absorb water, which occurs rapidly and continuously during the first 90 min (Figure 6). The neat nanocellulose CMNC has a swelling capacity of roughly 16,000%, which is considerably larger than the swelling capacity of e.g. acrylamide or acrylamide-bacterial cellulose hydrogels.^17,41^ Note that it was not possible to measure the swelling capacity of the CMPNC sample, as the freeze-dried structures easily disintegrated when they came in contact with water. In the CMPNC sample, the OH-groups in the C2 and C3 positions are replaced by aldehyde groups, while the OH-group in the C6 position is ether-bonded to a carboxymethyl group. Such a surface modification reduces the amount of OH groups, which are necessary for forming hydrogen bonds between adjacent nanofibrils during drying.

In order to demonstrate a cross-linking approach by ionic links, the CMNC and CMPNC gels were formed in a surplus of Ca^2+^. Considering that the hydrogels CMNC and CMPNC are immersed in water with neutral pH, the carboxyl groups are ionized having a COO^−^ form. At this point, it is worth to mention that the p*K*_a_ of carboxyl groups has been assumed to be 4.8,^2^ which explains the ionization at neutral pH. The anions create anionic bonds with the Ca^2+^, thus partly stabilizing the gel. Contrary to the neat CMPNC, the Ca^2+^-CMPNC composite gel was thus capable of maintaining a solid structure, which could be immersed, lifted and weighed several times. The swelling of the Ca^2+^-CMPNC was lower than CMNC samples probably due to the lower amount of OH-groups in the cellulose polymer.

It is worth to mention that the oxidized state of the cellulose nanofibrils could be applied to form Ca^2+^-nanocellulose beads analogous to Ca^2+^-alginate beads, which have been proposed for a range of biotechnological applications.^42^ This approach is promising and will thus widen the applicability of nanocellulose as a biomaterial.

### pH-responsive hydrogels

As mentioned above, the p*K*_a_ of carboxyl groups has been assumed to be 4.8.^2^ At lower values than the p*K*_a_, the carboxyl groups are expected to be in their COOH form. At higher pH, the COO- predominates allows for stronger ionic repulsion between the nanofibrils, i.e. the hydrogel swells.

The liquid absorption depends on the pH of the liquid. This capability is due to the swelling property of oxidized nanocellulose hydrogels, which have ionisable functional groups. This also explains the lower swelling capacity of the Ca^2+^-nanocellulose structures (Figures 5 and 6), due to the ionic interactions between the carboxyl groups and the divalent cations. The ionic interactions reduce the repulsive forces between the anionic groups.

The swelling capacity of the material in varying pH is most interesting as the responsive characteristics of the hydrogels can be used for controlled release of e.g. antibacterial and antibiofilm agents. Studies have demonstrated a relationship between the pH in wounds and the corresponding rate of healing. Chronic non-healing wounds have elevated alkaline environment, with pH between 7.15 and 8.9.^43^ As healing progresses, the pH becomes acidic. Acidic pH seems thus to be beneficial in wound management. As an example, acetic acid has been applied in the treatment of wound infections for the elimination of *Pseudomonas aeruginosa*.^44^

**Figure 7.**
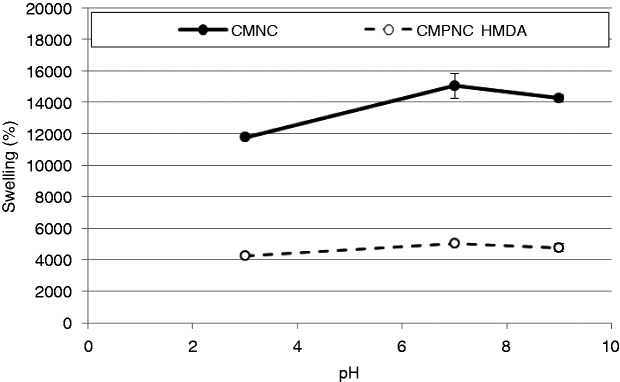
Water absorption as a function of pH. The CMPNC sample was cross-linked with hexamethylenediamine (HMDA). The results are given with ±1 STD.

The measurement of the water absorption exemplifies the pH-responsive capabilities of the manufactured hydrogels. Considering the CMNC sample, at low pH, the gel has a lower swelling compared to neutral and alkaline pH. The same trend is observed with the cross-linked HMDA–CMPNC hydrogel. However, the swelling of the HMDA–CMPNC is considerably lower, compared to the neat CMNC sample (Figure 7).

The hydrogel swelling is lower at pH 9 compared to pH 7 due to the larger concentrations of salt, which leads to charge shielding of the carboxylate ions.^45^ The ionic strength is thus lower in the neutral swelling medium, compared to the alkaline and acidic media.^17^

As mentioned above, the micro-porous and pH-responsive hydrogels can be applied for wound-healing applications, due to the capacity of the material to keep a moist environment and to the possibility of functionalizing the material with additional components, which could promote healing. In addition, the pH-responsive characteristics of the hydrogels could potentially be exploited for biosensing capabilities, i.e. monitoring a healing process without removing the wound dressing from the wound bed.

## Conclusions

In this study, we demonstrated the production of nanocellulose with tailor-made surface chemistry, by pre-treating the cellulose fibres with carboxymethylation and periodate oxidation. The pre-treatment yielded a highly fibrillated material, with significant amounts of aldehyde and carboxyl groups. It is suggested that the poly-anionic characteristics of oxidized nanocellulose can open up for novel applications, i.e. micro-porous materials with pH-responsive characteristics. This is due to the swelling property of the nanocellulose structures, which have ionisable functional groups. We demonstrated that nanocellulose gels have a significantly higher swelling degree in neutral and alkaline conditions, compared to an acid environment (pH 3). Such a capability could potentially be applied in the management of chronic wounds for controlled and intelligent release of antibacterial components into biofilms.
